# Antibacterial, Antibiofilm, and Antioxidant Activity of Polysaccharides Obtained from Fresh Sarcotesta of *Ginkgo biloba*: Bioactive Polysaccharide that Can Be Exploited as a Novel Biocontrol Agent

**DOI:** 10.1155/2021/5518403

**Published:** 2021-06-15

**Authors:** Hong Jiang, Zuxiang Luan, Zhaobing Fan, Xinliang Wu, Ziheng Xu, Tiezhong Zhou, Hongjun Wang

**Affiliations:** ^1^Institute of Animal Husbandry and Veterinary Medicine, Jinzhou Medical University, Jinzhou 121001, Liaoning, China; ^2^School of Teacher Education, Nanning Normal University, Nanning 530001, Guangxi, China; ^3^College of Pharmacy, Heze University, Heze 274015, Shandong, China; ^4^Department of Pharmacy, Tianjin Baodi Hospital, Baodi Clinical College, Tianjin Medical University, Tianjin 301800, China; ^5^Institute for Poultry Science and Health, Guangxi University, Nanning 530004, Guangxi, China

## Abstract

*Staphylococcus aureus* (*S. aureus*) biofilm plays an important role in the persistence of chronic infection due to its resistance to antibiotics. Because of their functional diversity, active polysaccharide is increasingly being applied as a biocontrol agent to inhibit the formation of biofilm by pathogens. In this study, a new polysaccharide, GBSPII-1, isolated from the fresh sarcotesta of *Ginkgo biloba L*. (*G. biloba*) was characterized and its effect on antibiofilm formation of *S. aureus* was examined *in vitro*. High-Performance Liquid Chromatography (HPLC) analysis showed that GBSPII-1 is an acidic heteropolysaccharide composed of mannose, rhamnose, glucose, glucuronic acid, and galacturonic acid. GBSPII-1 demonstrated a molecular weight of 34 kDa and may affect the accumulation of polysaccharide intercellular adhesion (PIA) by inhibiting *ica*A, *ica*B, *ica*C, and *ica*D gene expression at subinhibitory concentrations. Under 10 g/L, GBSPII-1 showed an antioxidant effect on the inhibition rate of H_2_O_2_-induced erythrocyte hemolysis and the scavenging rate of DPPH radicals was 76.5 ± 0.5% and 89.2 ± 0.26%, respectively. The findings obtained in this study indicate that GBSPII-1 has antibacterial effect, is a possible source of natural antioxidants, and may be a potential biocontrol agent for the design of new therapeutic strategies for biofilm-related *S. aureus* infections.

## 1. Introduction

*Staphylococcus aureus* (*S. aureus*) is the most common pathogen responsible for various suppurative diseases such as bacteraemia, endocarditis, food poisoning, pneumonia, sepsis, and cow mastitis [1–3]. *S. aureus* is also involved in a variety of biofilm-related infections such as those associated with heart implants, bloodline catheters, superficial skin infections, and otitis media [1, 2]. *S. aureus* can also act as a primary opportunistic pathogen. *S. aureus* infections are mostly treated with chemical drugs, but the emergence of methicillin-resistant *S. aureus* (MRSA) has become a serious global medical problem for the treatment of *S. aureus* infections because most clinical MRSA strains show high multidrug resistance [4, 5]. The term biofilm refers to a group of organized bacteria that adhere to almost every surface and are surrounded by extracellular bacterial macromolecules [6]. Within biofilms, there is a constant exchange of matter, energy, and information within cells, between cells, and between cells and the external environment, so that the life process can be carried out in a coordinated and orderly manner [7, 8]. The bacteria in the biofilm can be ejected at any time and participate in the formation of another biofilm, or infect the host and cause secondary infection and severe drug resistance [9]. The treatment of biofilm infections is extremely difficult as biofilm bacteria are highly resistant to antibiotics and host immune defense mechanisms. The formation of the biofilm multicellular structure is a dynamic process, which includes the initial adhesion of bacteria, biofilm development, and maturation. Biofilm not only exists as a barrier to cell life activities to create a stable internal environment and cell mediated by cell and the connection between the matrix but also allows transmembrane transport, information transfer, and energy conversion [3]. In addition, *S. aureus* biofilms pose a serious clinical threat as reservoirs for persistent infections. Unlike planktonic bacteria, biofilms associated *S. aureus* shows strong resistance to fungicides and the host immune defense mechanisms; the bacteria in the biofilm have a unique physiology, metabolism, pathogenicity, and resistance to environmental threats and can lead to recurrent infection and increase fatalities [10, 11]. Thus, it is important to identify new therapeutic strategies that can control biofilm development.

The development of a *S. aureus* biofilm is mainly facilitated by polysaccharide intercellular adhesion (PIA), a polysaccharide composed of *β*-1, 6-linked N-acetylglucosamine with partially deacetylated residues; PIA production leads to elaborating multilayered cell clustering. PIA surrounds the bacteria and protects them against both host immune defenses and antibiotic treatment [12]. The *ica*A, *ica*B, *ica*C, and *ica*D operon encode the proteins necessary for the synthesis of PIA and capsular polysaccharide/adhesion (PS/A) in staphylococcal species [13]. The products of the *ica*A, *ica*C, and *ica*D are located in the membrane fraction, while the product of the *ica*B gene is mainly found in the culture supernatant and deacetylates PIA when it is localized on the cell surface [14, 15]. Coexpression of *ica*A and *ica*D increases N-acetylglucosaminyl transferase activity and slime production [16]. Thus, PIA is an important factor in the *S. aureus* biofilm formation.

*Ginkgo biloba L*. (*G. biloba*) is an endemic species of China that has important pharmacological activities, including immunity-enhancing activity, antianaphylaxis effect, antibacterial effect, and antiaging activity [17], and is well known as a “living plant fossil.” However, the sarcotesta of *G. biloba* was previously regarded as a waste material. Phenolic acid, found in the discarded sarcotesta of *G. biloba*, can change soil composition and water quality, not only contaminating soil but also poisoning fish and shrimp [17]. The sarcotesta of *G. biloba* also has a pungent odor, which causes air pollution. The polysaccharide composition is 10% in the sarcotesta of *G. biloba* [18]. Crude polysaccharide is the yellow-brown colored powder, odorless, and slightly sweet and has no toxic side effects on the human and animal body. Particularly, polysaccharide extracted from the dry sarcotesta of *G. biloba* (GBSP) is an active polysaccharide that shows immunity-enhancing activity, antitumor effect, and antioxidant activity [19–21].

In order to seek more effective and environmentally friendly bioactive molecules, GBSP was extracted from the fresh sarcotesta of *G. biloba*, and the antibacterial, antibiofilm, and antioxidant activity test was conducted *in vitro*. Further, we also studied the expression levels of PIA, recognized as one of the determining factors in *S. aureus* biofilm formation. The expression of *ica*A, *ica*B, *ica*C, and *ica*D genes was also studied.

## 2. Materials and Methods

### 2.1. Bacterial Strains, Growth Conditions, and Materials

The fresh sarcotestas of *G. Biloba* were obtained from a *G. Biloba* tree in the garden of the Jinzhou Medical University (Jinzhou, China) from September to October 2016. GBSPII-1 was then extracted and isolated from the fresh sarcotestas of *G. Biloba* in the animal pharmaceutical laboratory of Jinzhou Medical University (Jinzhou, China). *S. aureus* (CVCC1882) was provided by the Chinese veterinary drug supervision (Beijing China). The strains were subcultured in brain heart infusion (BHI) solution (Oxoid, Basingstoke, UK) that contained 3% NaCl and 0.5% glucose and were incubated for 12 h at 37°C. *S. aureus* CVCC1882 was grown in enriched tryptic soy broth supplemented with 1% glucose (TSBg; Merck, Darmstadt, Germany Baker, UK) and for all experiments, bacterial cultures were grown aerobically in 6-well plates (Costar3595; Corning Life Sciences) at 37°C. Sytox green, fluorescein isothiocyanate (FITC), Syto 63, Congo red, and crystal violet were obtained from Tianjin Heowns Biochemical Technology Co., Ltd. (Tianjin China).

### 2.2. Monosaccharide Composition Analysis of GBSPII-1 by HPLC

Monosaccharide composition analysis of GBSPII-1 was assessed by HPLC precolumn derivatization as previously described [22]. Briefly, reference monosaccharides, including Ara, Glu, glucuronic acid, Gal, Rha, Man, and galacturonic acid, or the samples were hydrolyzed with trifluoroacetic acid (TFA, 2 M) in a 110°C drying box. The hydrolyzed GBSPII-1 or reference monosaccharide solution was derivatized with PMP (0.5 M) in methanol and 0.3 M NaOH solution in a 75°C water bath. These derivatives were analyzed by HPLC (Hitachi Ltd., Japan).

### 2.3. Determination of the Molecular Weight of GBSPII-1

The molecular weight of GBSPII-1 was determined according to a previously reported method [23]. Briefly, six molecular weight reference glucans (T-10, T-20, T-40, T-70, T-110, and T-500) (Sigma, MO, USA) and the GBSPII-1 test sample were loaded onto TSK-Gel G2000PW columns (TOSOH Company, Japan) and run on a Hitachi HPLC pump system (HITACHI, Japan) equipped with a refractive index detector (Shodex RI SE-51, Japan). The distribution coefficient (Kav) and logarithmic molecular weight of six reference glucans (logM) allowed the generation of a standard curve. The molecular weight of GBSPII-1 was then calculated based on the standard curve.

### 2.4. Antibacterial Activity Assays

The minimum inhibitory concentration (MIC) of GBSPII-1 was determined by a microtiter broth dilution plate method, followed the CLSI guidelines but with some modifications for natural products [23–25]. In brief, the BHI broth was used to serial dilute GBSPII-1 two-fold, and S. aureus CVCC1882 was added at a concentration of 1 × 105 CFU/mL to 96-well plates (Costar 3595; Corning Life Sciences). After the plates were incubated at 37°C for 24 h, the culture in each well was applied to the AGAR plate and incubated at 37°C for 12 h again. And the *Escherichia coli* (ATCC 25922) was used as a quality control organism.

### 2.5. Crystal Violet Screening Assay for Biofilm Formation Time

Overnight *S. aureus* cultures were diluted 1 : 100 with fresh BHI containing 3% NaCl and 0.5% glucose. Then, 200 *μ*L cultures were transferred into 96-well plates and incubated at 37°C for 2, 4, 8, 12, 16, 24, and 48 h, respectively. The cultures were removed and every well was washed twice with sterile phosphate-buffered saline (PBS) solution at pH 7.4 to remove nonadherent cells and dried in an inverted position. The adhesive bacteria were fixed with methanol and stained with 1% crystal violet (Tianjin Heowns Biochemical Technology Co. Ltd., China) for 5 min. The excess stain was poured out and the wells were washed three times with sterile distilled water. The water was then removed and the microplates were air-dried. To every well, 200 *μ*L ethanol was added to dissolve dyed adherent bacteria and 10-fold dilutions of the dissolved liquid in ethanol. The optical density (OD) of each well was measured at 570 nm (OD_570_) using an ultraviolet spectrophotometer (Shanghai Meipuda Instrument Co. Ltd., China). The OD value reflects bacterial adhesion to the contact surface and biofilm formation [26].

### 2.6. Biofilm Eradication Assay

*S. aureus* was grown in 5 mL TSB containing 0.25% glucose (TSBg) at 37°C for 24 h. A total of 10 mL (10^4^ CFU) *S. aureus* was equally distributed into each well of the 96-well plates containing TSBg. The wells containing only TSBg were used as blank controls. Then, plates were incubated at 37°C for an appropriate time to allow biofilm development. Each experiment was tested in triplicate. After incubation, the culture medium was discarded from each well and wells were washed three times with sterile PBS solution. The different concentrations of the tested GBSPII-1 (100, 50, and 25 g/L) were then added to each well. In order to evaluate the biomass of the biofilm, the plates were further incubated for 24 h, and then a crystal violet assay was used to determine the effect of GBSPII-1 on biofilm formation according to a previously described method [27, 28].

### 2.7. Confocal Microscopy Assay

As described above, the biofilm was formed on glass [29]. The formation of *S. aureus* biofilm was evaluated by confocal laser-scanning microscopy (CLSM) (Olympus, Shanghai, China). *S. aureus* was incubated in 96-well plates at 37°C for 12 or 24 h. After culturing, the wells were washed twice with 2 mL of PBS. PBST (PBS contains 0.5% Triton x-100) containing Sytox green (0.5 aerobic) was added to the 96-well plates and shaken for 30 min. Then, PBS containing FITC (0.001%) and Syto 63 (100 *μ*M) was added to the plates, which could not be shaken. The supernatants of the wells were removed and were washed three times with PBS. Confocal microscope images were analyzed using Fluoview version 1.7.3.0 software in SP5II CLSM.

### 2.8. RNA Isolation from Biofilms

One milliliter of logarithmic phase *S. aureus* and *S. aureus* treated with the different GBSPII-1 concentrations (1/2 MIC, 1/4 MIC, 1/8 MIC) was taken and RNA was isolated from *S. aureus* using the total RNA rapid extraction kit (Shanghai Shengong Biological Engineering Co. Ltd., China), according to the manufacturer's instructions and a previously described method [30]. In brief, after the culture medium was completely removed, 100 *μ*L 3 mg/mL lysozyme was added for enzymolysis for 5–10 min at room temperature. The enzymolysis products were washed once with cold sterile DEPC-treated ddH_2_O. The supernatant was discarded and products centrifuged at 10,000 rpm for 1 min. Rlysis-B buffer was immediately added to the precipitates and the mixture oscillated for 3 min at room temperature. Then, the RNA was extracted from the mixture by the addition of chloroform and ethanol. The RNA quality and quantity were determined by agarose gel electrophoresis. Residual DNA present in the RNA preparation was removed using RNase-free DNase I and gDNA wipeout buffer (Shengong). Further, total RNA samples were analyzed for the presence of DNA contamination by PCR targeting the 16S rRNA gene without the reverse transcriptase step (no cDNA) [31], using the same conditions as for qPCR. Purified RNA was immediately converted to cDNA to avoid RNA degradation using RevertAid™ First Strand cDNA Synthesis Kit (Hangzhou Bori Technology Co. Ltd., China) with random hexamer primers according to the manufacturer's instructions (Table 1).

### 2.9. Primers Specificities for qPCR and Quantitative Real-Time PCR

As described in the previous literature, the *ica* series target gene and reference gene primers were synthesized by Bioengineering (Shanghai) Biology Company (Table 1) [31]. After transcriptional cDNA, the specificity of 16S rRNA, *ica*A, *ica*B, *ica*C, and *ica*D gene primers was verified by standard PCR. The identities of all PCR products were confirmed by sequencing and the amplification efficiency for each primer set was determined by a RT-qPCR assay. The reaction was carried out in ABI 7500 Fast Real-Time PCR Instrument (ABI, USA). Reactions were performed in triplicate using 96-well plates and the reaction volume was set at 20 *μ*L per sample. All reactions contained 1 *μ*L of cDNA, 10 *μ*L of 2 × SuperReal PreMix Plus, 0.6 *μ*L of each primer, 0.5 *μ*L of 50 × ROX Reference Dye, and 7.3 *μ*L of sterile double RNase treated water. The reaction was started with an initial denaturation at 95°C for 15 min and 40 amplification cycles of 95°C for 10 s and 60°C for 30 s.

### 2.10. Antioxidant Activity

#### 2.10.1. DPPH Radical Scavenging Assay

The DPPH free radical scavenging activity of GBSPII-1 was evaluated as previously described [19]. Briefly, GBSPII-1 was dissolved and diluted to five concentrations of 0.1–20 g/L in water. Vitamin C (VC) was used as a positive control and diluted to a concentration of 1.0 g/L using ethanol. DPPH was reacted with the GBSPII-1 sample solutions and the positive controls in ethanol. After 30 min, the absorbance values were measured at 517 nm using a UV-757CRT spectrophotometer (Shanghai Precision Instrument Co., Ltd., in China) and the DPPH free radical scavenging rate was calculated using the formula.

#### 2.10.2. Oxidative Hemolytic Inhibitory Activity

The inhibitory activity of H_2_O_2_-induced hemolysis of red blood cells was determined as previously described, with some modification [19]. Briefly, red blood cells of mice and H_2_O_2_ were mixed with 0.4 mL of GBSPII-1 (0.1–20 g/L) and VC (1.0 g/L) for 1 h at 37°C. Then, the resulting samples were diluted 6-fold with normal saline (NS), centrifuged for 10 min at 2000 rpm and the absorbance value of the supernatant was measured at 415 nm against a blank. The scavenging ability of the hydroxyl radical was calculated using the formula.

### 2.11. Statistical Analysis

Student's *t*-test was performed with version 19.0 of IBM SPSS software to assess biofilm-forming capacity, relative gene expression, and antioxidant activity. The data were expressed as the mean ± standard deviation. Results with values of *p* < 0.05 and *p* < 0.01 were considered statistically significant.

## 3. Results

### 3.1. Monosaccharide Composition of GBSPII-1

**Figure 1:**
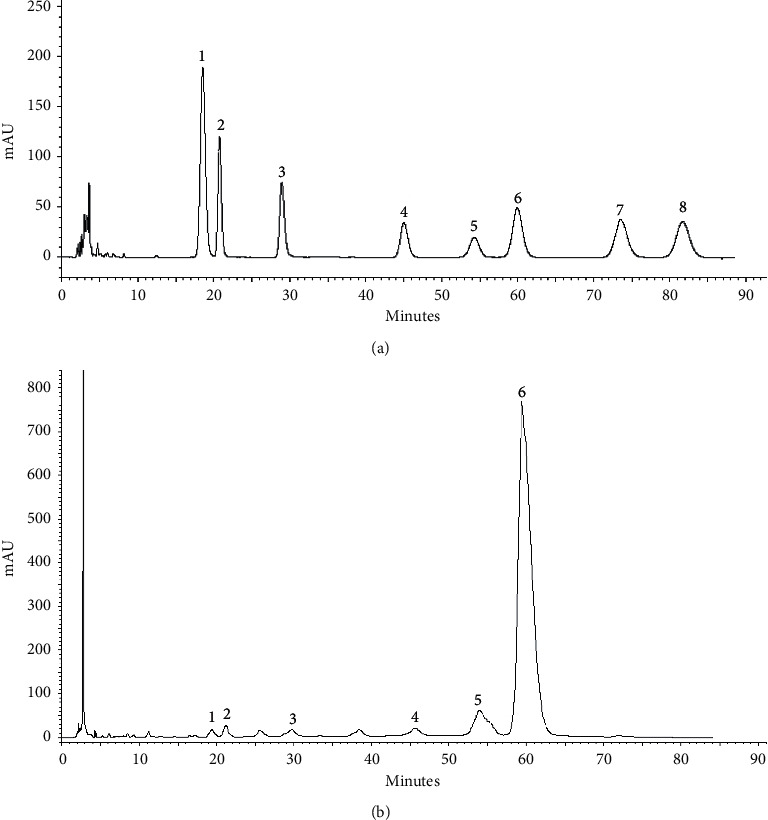
Eight reference monosaccharides analysis (a) and monosaccharides compositions analysis of GBSPII-1 by HPLC precolumn derivatization (b). 1 represents PMP; 2 represents d-mannose (Man); 3 represents d-rhamnose (Rha); 4 represents glucuronic acid; 5 represents galacturonic acid; 6 represents d-glucose (Glu); 7 represents d-galactose (Gal); and 8 represents d-arabinose (Ara).

According to the results (Figure 1(a) and Figure 1(b)), GBSPII-1 is composed of Man, Rha, Glu, glucuronic acid, galacturonic acid, and two unknown sugars. The composition of GBSPII-1 was composed of Man, Rha, glucuronic acid, glucuronic acid, and Glu in a molar ratio of 0.15 : 0.11 : 0.13 : 0.82 : 4.94.

### 3.2. Molecular Weight of GBSPII-1

**Figure 2:**
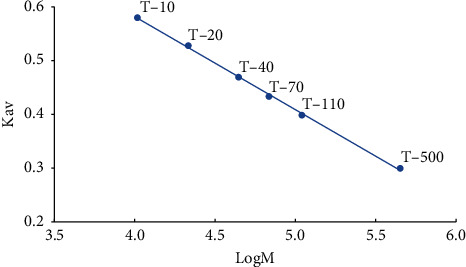
Linear curve of logM and Kav. Six glucans (T-10, T-20, T-40, T-70, T-110, and T-500) were used as standards and run on HPLC to generate a standard curve using Microsoft office excel 2016.

Our results showed that the average retention time of blue glucan-2000 (Vo) was 22.02 ± 0.32 min (*n* = 3) and that the average retention time of glucose (Vt) was 42.36 ± 0.24 min (*n* = 3). A standard curve (Figure 2) was generated using Microsoft Excel 2016 to analyze the data acquired. The linear regression equation Kav = 1.274–0.173 logM, *R*^2^ = 0.9989, was also obtained. For the GBSPII-1 samples, the average retention time was 32.00 ± 0.09 min; using the two known equations, the molecular weight was thus calculated to be 33,688 Da (approximately equal to 34 kDa).

### 3.3. GBSPII-1 Antibiofilm Formation with Affecting Planktonic *S. aureus* CVCC1882 Growth

**Figure 3:**
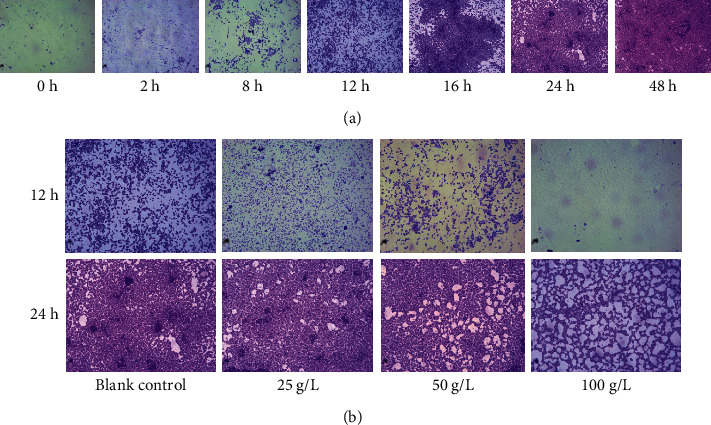
*S. aureus* biofilm formation was determined by crystal violet staining and microscopic observation (1000x). (a) *S. aureus* biofilm formation at 0 h to 48 h range. (b) The antibiofilm activities of GBSPII-1 against *S. aureus* were determined.

The MIC of GBSPII-1 was 1.563 g/L against planktonic *S. aureus* CVCC1882 growth. Minimum biofilm inhibitory concentration (MBIC) and minimum biofilm bactericidal concentration (MBBC) of GBSPII-1 were both 100 g/L. We conducted a screening of phytochemicals with antibiofilm activity against *S. aureus* in 96-well polystyrene plates. After incubation for 12 h, *S. aureus* adheres to solid surfaces to form biofilms (Figure 3(a)). Different concentrations of GBSPII-1 inhibited *S. aureus* biofilm formation at 12 and 24 h (Figure 3(b)). The results showed that 100 g/L GBSPII-1 markedly inhibited *S. aureus* biofilm formation at 12 h (Figure 3(b)), but it could not inhibit *S. aureus* biofilm formation at 24 h (Figure 3(b)). Therefore, we studied the action mode and matrix composition of GBSPII-1 at the stage of biofilm development. We found that GBSPII-1 terminated the growth process of biofilms when added in the initial adhesion and proliferation stage.

**Figure 4:**
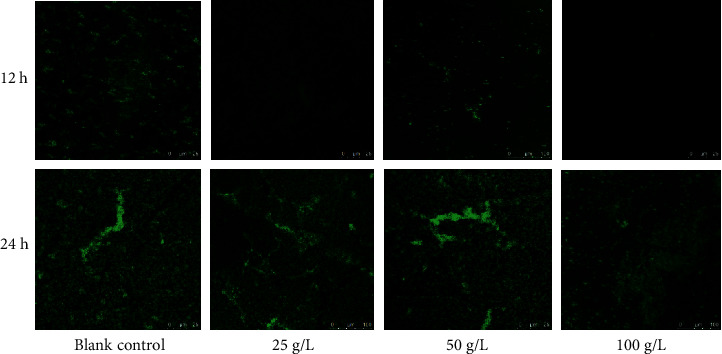
*S. aureus* CVCC1882 was incubated with different concentrations of GBSPII-1. Biofilm formation on glass was observed at 12 h and 24 h, respectively, by confocal laser microscopy. Scale bar represents 20 *μ*m.

CLSM was used to analyze changes in biofilm formation as shown in Figure 4. The fluorescent images indicated that, in the control group, the biofilms are gradually increased at 12 h and 24 h and GBSPII-1 dose-dependently inhibited *S. aureus* CVCC1882 biofilm formation. The biofilm formed significant clumps at 24 h. GBSPII-1 could inhibit the formation of early biofilm. With the increase of GBSPII-1 concentration, the green fluorescence intensity also decreased, and the number of living bacteria decreased in 12 h. GBSPII-1 had a lesser inhibitory effect at 24 h. The results indicated that GBSPII-1 had a greater inhibitory effect on early biofilm than mature stage biofilm.

### 3.4. Expression Levels of PIA Regulatory Gene Quantified by qPCR

**Figure 5:**
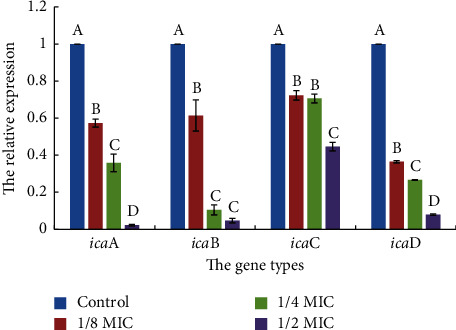
The relative expression levels of 4 adhesion and biofilm genes in response to different concentrations of GBSPII-1 incubation in *S. aureus* CVCC1882. Total RNA was isolated from 12 h old cultures of GBSPII-1 incubation in *S. aureus* CVCC1882, and the relative expression levels of *ica*A, *ica*B, *ica*C, and *ica*D genes were compared by real-time RT-PCR (relative plotted against log10). The relative expression levels of these genes were calculated at different concentrations of GBSPII-1 incubation with *S. aureus* CVCC1882. Data are representative of three independent experiments. The expression of *ica*A, *ica*B, *ica*C, and *ica*D genes was significantly inhibited by GBSPII-1. Uppercase letters represent extremely significant difference of *p* < 0.01 compared with the control group.

The expression levels of 4 genes involved in biofilm formation, *ica*A, *ica*B, *ica*C, and *ica*D, were compared using Ct values, which showed significant differences at different concentrations of GBSPII-1 (mean Ct values ranging from 26.42 to 36.74). The concentrations of GBSPII-1 ranging from 1/8MIC to 1/2MIC can inhibit the expression of 4 selected genes (Figure 5). The comparative relative expression of 4 selected genes at each different concentration of biofilm formation for *S. aureus* CVCC1882 isolates was calculated relative to the reference gene in Table 2. When the concentration of GBSPII-1 is 1/2 MIC (0.78 g/L), GBSPII-1 significantly inhibits the expression of *ica*A, *ica*B, and *ica*D genes (*p* < 0.05). The relative expressions of *ica*ABD gene are reduced by 90%, while the expression of *ica*C gene is reduced by 55%. With the concentration of the GBSPII-1 for 1/4 MIC (0.395 g/L), downregulated *ica*B gene expression is not a significant difference compared with 1/2 MIC GBSPII-1 group (*p* > 0.05) and downregulated *ica*C gene expression is not a significant difference compared with 1/8 MIC GBSPII-1 group (*p* > 0.05). When GBSPII-1 concentration was 1/8 MIC, *ica*D gene expression was reduced by 60%.

REF indicates reference gene; TRG indicates target gene, *ica*A, *ica*B, *ica*C, and *ica*D, to intercellular adhesion biofilm required genes. Black downward arrows indicate being significantly decreased if *p* value is <0.05 (target sample is different from control).

### 3.5. Antioxidant Activity In Vitro

**Figure 6:**
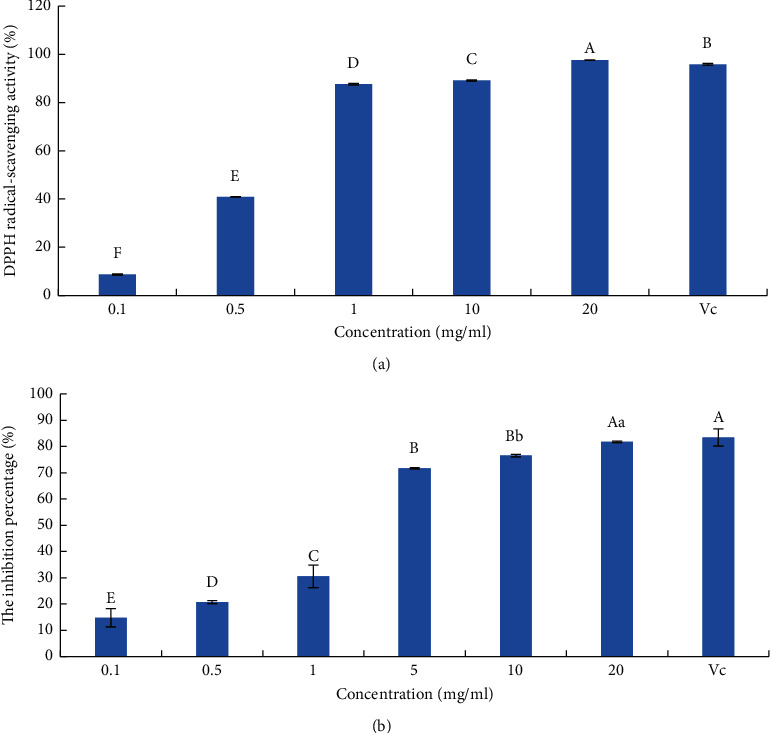
Antioxidant activity of the tested GBSPII-1. (a) DPPH assay. (b) Oxidative hemolysis inhibitory assay. VC: 1.0 g/L vitamin C for each mean; the respective standard deviation is shown (mean ± SD). Uppercase letters represent an extremely significant difference of *p* < 0.01; lowercase letters represent a significant difference of *p* < 0.05.

As shown in Figure 6(a), GBSPII-1 has a concentration dependence on DPPH free radical scavenging rate. Therefore, GBSPII-1 showed a promising antioxidant potential. At a concentration of 1.0 g/L, the scavenging rates were 87.65% and 95.87% for GBSPII-1 and the VC (1.0 g/L) positive control group, respectively. The DPPH scavenging rates of 20 mg/mL GBSPII-1 were significantly higher than those of VC (*p* < 0.01). Otherwise, GBSPII-1 showed a concentration-dependent inhibition of H_2_O_2_-induced hemolysis in mice red blood cells (Figure 6(b)). As the concentration of GBSPII-1 increased from 0.5 g/L to 20 g/L, the inhibition rate increased from 20.72% to 81.72%. Therefore, the inhibition rate of hemolysis significantly increased with increasing GBSPII-1 concentrations (*p* < 0.05 or *p* < 0.01). Compared with the VC positive control group, there was no significant difference in inhibition rate of hemolysis between 20 g/L GBSPII-1 and the control group (*p* > 0.05).

## 4. Discussion

The polysaccharides have many bioactive functions, including antibacterial activity. In this study, GBSPII-1 is composed of Man, Rha, Glu, glucuronic acid, galacturonic acid, and two unknown sugars. This polysaccharide is different from previous reports [19], indicating that GBSPII-1 is a new polysaccharide isolated from the fresh sarcotesta of *Ginkgo biloba*. Further studies on the elucidation of the biological functions of the novel polysaccharide are underway. So, we evaluated the inhibitory effect of GBSPII-1 on *S. aureus* with a MIC value of 1.563 mg/mL, which is superior to most plant polysaccharides. This may be related to the fact that our polysaccharides are extracted from fresh sarcotesta of *G. biloba*. The antibacterial mechanism of plant polysaccharides is complex and needs to be further clarified [32–35]. However, some studies have shown that polysaccharides can improve the number and function of T lymphocytes and macrophages in vivo and then enhance the immune defense function of the body to inhibit the bacterial growth [33, 34]. In vitro, the direct inhibition and detoxification effect of polysaccharides on bacteria and their toxic products may be due to the antimicrobial substances contained by polysaccharides such as aldehyde acid and some structures that recognize certain bacterial binding protein receptors contained by polysaccharides, which can inhibit all stages of bacterial metabolism such as protein metabolism and glucose metabolism and inhibit the growth of bacteria by destroying the morphology of bacterial cells [36]. In this study, GBSPII-1 has strong antibacterial activity, indicating that GBSPII-1 with antibacterial biological activity could be developed as biological control agents.

*S. aureus* is of great concern because of its widespread spread and high drug resistance. It has been reported that super drug-resistant bacteria, methicillin-resistant *S. aureus* and vancomycin-resistant *S. aureus*, appeared clinically probably due to their ability to form biofilms in medical devices [4, 31, 37–39]. Bacteria living in a biofilm tend to be resistant to antibiotics and thus require higher doses. The results of this study showed that the MIC value of GBSPII-1 on *S. aureus* was 1.563 mg/mL, while the MBIC and MBBC of GBSPII-1 on *S. aureus* biofilm were 100 mg/mL. And the concentration of the polysaccharide was increased significantly nearly 60 times, verifying that *S. aureus* greatly improved bacterial resistance due to the protection of biofilm. The biofilm infection problems are complex and common, especially medical devices infection causing clinical failure and impaired functionality, as well as reducing the lifetime of medical devices [38, 39]. So, it is very important to control biofilm formation. In this study, the growth state of *S. aureus* biofilm at different times was detected, which can effectively judge the time point of biofilm formation of *S. aureus* in early and mature stages. At present, after the biofilm formation, most plant extracts have no obvious inhibitory effect on it. Because they encapsulate bacteria at the maturity stage and have strong drug resistance. The inhibitory effect of the GBSPII-1 on the *S. aureus* biofilm in the early and mature stages was detected, obtaining the GBSPII-1 concentration of the biofilm in different periods. But some bacteria will break away from the biofilm after the biofilm formation, which can prevent further infection of *S. aureus*. Therefore, this study provided a theoretical basis for the development of inhibitors of *S. aureus* biofilm and the prevention of further infection of the *S. aureus* biofilm.

The mechanism of biofilm is complex, but the key pathway is PIA secretion in the early stages. PIA is the product of *ica*A, *ica*D, *ica*B, and *ica*C genes expression regulated by *ica* operons [31]. The protein expressed by the genes of *ica*A, *ica*D, and *ica*C is located in the membrane. And *ica*B gene encodes an extracellular protein, while *ica*C encodes a membrane protein that is currently thought to have a receptor for the polysaccharide [31]. At present, the inhibition effects of the drug on the expression of genes related to *S. aureus* biofilm are mainly studied on the transcription level of *ica*A gene. Studies have shown that the tea tree oil with a concentration of 0.5 mg/mL can inhibit the expression of 90% of *ica*A gene and can effectively inhibit the formation of biofilm [37]. However, this does not fully explain the mechanism of drug inhibition on PIA, nor does it explain the mechanism of inhibition on the biofilm formation. In this study, the effects of GBSPII-1 on the expression of *ica*A, *ica*D, *ica*B, and *ica*C genes were studied, and it was found that the GBSPII-1 with different subinhibitory concentrations had a certain inhibitory effect on these genes and was dose-dependent. Among them, GBSPII-1 with half MIC (0.78 mg/mL) concentrations inhibited the *ica*A gene expression by 97%, which was better than that of tea tree oil. The GBSPII-1 has a good inhibitory effect on the expression of the four genes, so the bacteriostatic mechanism, where GBSPII-1 inhibits the expression of related genes, thus inhibiting the formation of proteins or destroying protein structures, reducing the secretion of PIA, and then inhibiting the formation of biofilm and the growth of bacteria, can be speculated [39]. However, the formation process of the biofilm is complex and is regulated by a variety of genes. GBSPII-1 may inhibit the formation of the biofilm of *S. aureus* through other pathways, and further in-depth research is needed.

The free radical attack can cause oxidative damage to biological macromolecules, such as nucleic acid (DNA, RNA), enzyme, protein, and sugar and affect the morphological function of the organ and immune system [40]. Research showed that hydroxyl radicals can react with DNA bases, causing DNA chain breakage and base damage [41]. Scavenging hydroxyl radicals are of great significance to the protection of DNA. When oxygen free radicals increase, red blood cells will undergo hemolysis, which will lead to the transformation of oxygen-carrying hemoglobin into methemoglobin and, in severe cases, will lead to coma or even death [42]. GBSPII-1 can effectively scavenge free radicals and avoid oxidative hemolysis of red blood cells, thus protecting the normal physiological function of the body. Therefore, in this experiment, DPPH free radical, hydroxyl radical scavenging, and erythrocyte oxidative hemolysis inhibition were used to evaluate their antioxidant ability in vitro. It was found that, in the DPPH free radical scavenging experiment, the antioxidant ability of 1 mg/mL polysaccharide solution was the same as that of vitamin C of the same mass concentration, and it was better than that of the dryers [19]. The evaluation model is comprehensive and direct, which can correctly evaluate the antioxidant ability of polysaccharides in vitro and provide a reference for the development of polysaccharides into natural antioxidants.

## 5. Conclusion

In summary, GBSPII-1, which was derived from the fresh sarcotesta of *Ginkgo biloba L*, is composed of Man, Rha, Glu, glucuronic acid, and galacturonic acid, and its molecular mass is approximately 34 kDa. We believe that one of the mechanisms of GBSII-1 in controlling biofilm formation is by reduced PIA secretion by inhibiting the expression of the icaA, icaB, icaC, and icaD genes, which leads to the failure of S. aureus to form a biofilm at a 100 g/L dose of GBSPII-1. In addition, GBSPII-1 showed an antioxidant effect on the inhibition rate of H_2_O_2_-induced erythrocyte hemolysis and the scavenging rate of DPPH. The findings obtained in this study indicate that GBSPII-1 has an antibacterial effect, is a possible source of natural antioxidants, and may be a potential biocontrol agent for the design of new therapeutic strategies for biofilm-related *S. aureus* infections.

## Figures and Tables

**Table 1:** Target genes and reference gene primers used for qPCR.

Genes	Sequence of primers (5′-3′)	Accession numbers	Amplicon size (bp)
*ica*A (intercellular adhesion gene A)	Forward: 5′-GAGGTAAAGCCAACGCACTC-3′	AF086783	151
	Reverse: 5′-CCTGTAACCGCACCAAGTTT-3′		

*ica*B (intercellular adhesion gene B)	Forward: 5′-ATACCGGCGACTGGGTTTAT-3′	AF086783	140
	Reverse: 5′-TTGCAAATCGTGGGTATGTGT-3′		

*ica*C (intercellular adhesion gene C)	Forward: 5′-CTTGGGTATTTGCACGCATT-3′	AF086783	209
	Reverse: 5′-GCAATATCATGCCGACACCT-3′		

*ica*D (intercellular adhesion gene D)	Forward: 5′-ACCCAACGCTAAAATCATCG-3′	AF086783	211
	Reverse: 5′-GCGAAAATGCCCATAGTTTC-3′		

16S rRNA	Forward: 5′-GGGACCCGCACAAGCGGTGG-3′	L37597.1	191
	Reverse: 5′-GGGTTGCGCTCGTTGCGGGA-3′		

**Table 2:** Fold change in mRNA levels of adhesion and biofilm target genes in cultures of different *S. aureus* isolates grown at 1/8, 1/4, and 1/2 MIC.

Genes	Type	Fold change in level of biofilm genes		
		1/8 MIC	1/4 MIC	1/2 MIC
16S	REF	1.0	1.0	1.0
*ica*A	TRG	0.57 ↓	0.36 ↓	0.02 ↓
*ica*B	TRG	0.61 ↓	0.10 ↓	0.04 ↓
*ica*C	TRG	0.72 ↓	0.71 ↓	0.45 ↓
*ica*D	TRG	0.36 ↓	0.27 ↓	0.08 ↓

## Data Availability

The data used to support the findings of this study are available from the corresponding author upon request.

## References

[1] Thornton R. B., Wiertsema S. P., Kirkham L.-A. S. (2013). Neutrophil extracellular traps and bacterial biofilms in middle ear effusion of children with recurrent acute otitis media—a potential treatment target. *PLoS One*.

[2] Agarwal A., Singh K. P., Jain A. (2010). Medical significance and management of staphylococcal biofilm. *FEMS Immunology & Medical Microbiology*.

[3] Xiang H., Cao F., Ming D. (2017). Aloe-emodin inhibits *Staphylococcus aureus* biofilms and extracellular protein production at the initial adhesion stage of biofilm development. *Applied Microbiology and Biotechnology*.

[4] Deurenberg R. H., Vink C., Kalenic S., Friedrich A. W., Bruggeman C. A., Stobberingh E. E. (2007). The molecular evolution of methicillin-resistant *Staphylococcus aureus*. *Clinical Microbiology and Infection*.

[5] Oyama K., Kawada-Matsuo M., Oogai Y., Hayashi T., Nakamura N., Komatsuzawa H. (2016). Antibacterial effects of glycyrrhetinic acid and its derivatives on *Staphylococcus aureus*. *PLoS One*.

[6] Lopez D., Vlamakis H., Kolter R. (2010). Biofilms. *Cold Spring Harbor Perspectives in Biology*.

[7] Branda S. S., Vik Å., Friedman L., Kolter R. (2005). Biofilms: the matrix revisited. *Trends in Microbiology*.

[8] Hall-Stoodley L., Stoodley P. (2009). Evolving concepts in biofilm infections. *Cellular Microbiology*.

[9] Xu Z. H., Wang H. J., Luan Z. H., Jiang H., Wang P., Wang J. L. (2017). Identification of *Staphylococcus aureus* in biofilm formation and inhibition of polysaccharide from *Ginkgo biloba* sarcotesta on biofilm. *Chinese Veterinary Science*.

[10] Lewis K. (2010). Persister cells. *Annual Review of Microbiology*.

[11] Waters E. M., Rowe S. E., O’Gara J. P., Conlon B. P. (2016). Convergence of *Staphylococcus aureus* persister and biofilm research: can biofilms be defined as communities of adherent persister cells?. *PLoS Pathogens*.

[12] Götz F. (2002). Staphylococcus and biofilms. *Molecular Microbiology*.

[13] Chaieb K., Mahdouani K., Bakhrouf A. (2005). Detection of icaA and icaD loci by polymerase chain reaction and biofilm formation by staphylococcus epidermidis isolated from dialysate and needles in a dialysis unit. *Journal of Hospital Infection*.

[14] Gerke C., Kraft A., Süßmuth R., Schweitzer O., Götz F. (1998). Characterization of the N-acetylglucosaminyltransferase activity involved in the biosynthesis of the staphylococcus epidermidis polysaccharide intercellular adhesin. *Journal of Biological Chemistry*.

[15] Vuong C., Kocianova S., Voyich J. M. (2004). A crucial role for exopolysaccharide modification in bacterial biofilm formation, immune evasion, and virulence. *Journal of Biological Chemistry*.

[16] Arciola C. R., Campoccia D., Baldassarri L. (2006). Detection of biofilm formation in staphylococcus epidermidis from implant infections. comparison of a PCR-method that recognizes the presence ofica genes with two classic phenotypic methods. *Journal of Biomedical Materials Research Part A*.

[17] Zhao S. Q., Li F., Sun Y. M. (2000). Research progressor the exopleura of *Ginkgo bilobal*. *Journal of Wuhan Botanical Research*.

[18] Shen W. S., Liu Y., Chen X. Y. (2014). Optimization of microwave-assisted extraction condition of polysaccharide from the episperm of *Ginkgo biloba* using response surface analysis. *Chinese Wild Plant Resources*.

[19] Chen J. (2011). *Purificaiton, Component Analysis and Anti-Oxidant Activity of Ginkgo biloba Exocarp Polysaccharide*.

[20] Yang L. Q., Mao G. H., Wu X. Y., Fan Q. Y., Zhu X. H. (2009). Effects of the crude polysaccharides from *Ginkgo biloba* sarcotesta on immune function in mice. *Li Shi Zhen Medicine and Materia Medica Research*.

[21] Liu W., Xie L. X., Liu X., Wang Z. X. (2012). The effects of *Ginkgo biloba* exocarp polysaccharides on proliferation of human endometrial cancer cell HEC-1B. *Chinese Journal of Biochemical Pharmaceutics*.

[22] Wang H., Xu Z., Li X. (2017). Extraction, preliminary characterization and antioxidant properties of polysaccharides from the testa of *Salicornia herbacea*. *Carbohydrate Polymers*.

[23] Wang H., Deng X., Zhou T. (2013). The in vitro immunomodulatory activity of a polysaccharide isolated from *Kadsura marmorata*. *Carbohydrate Polymers*.

[24] Kim E.-S., Kang S.-Y., Kim Y.-H. (2015). *Chamaecyparis obtusa* essential oil inhibits methicillin-resistant *Staphylococcus aureus* biofilm formation and expression of virulence factors. *Journal of Medicinal Food*.

[25] Jiang H., Zhou T. Z., Li X. G. (2013). Antimicrobial activity of compound traditional Chinese medicine feizhu powder. *Advanced Materials Research*.

[26] Fox L. K., Zadoks R. N., Gaskins C. T. (2005). Biofilm production by *Staphylococcus aureus* associated with intramammary infection. *Veterinary Microbiology*.

[27] Chaieb K., Kouidhi B., Harzallah H. J., Mahdouani K., Bakhrouf A. (2011). Antibacterial activity of thymoquinone, an active principle of *Nigella sativa* and its potency to prevent bacterial biofilm formation. *BMC Complementary and Alternative Medicine*.

[28] Mahdhi A., Leban N., Chakroun I. (2017). Extracellular polysaccharide derived from potential probiotic strain with antioxidant and antibacterial activities as a prebiotic agent to control pathogenic bacterial biofilm formation. *Microbial Pathogenesis*.

[29] Hochbaum A. I., Kolodkin-Gal I., Foulston L., Kolter R., Aizenberg J., Losick R. (2011). Inhibitory effects of D-amino acids on *Staphylococcus aureus* biofilm development. *Journal of Bacteriology*.

[30] Atshan S. S., Shamsudin M. N., Lung L. T. T. (2012). Improved method for the isolation of RNA from bacteria refractory to disruption, including *S. aureus* producing biofilm. *Gene*.

[31] Atshan S. S., Shamsudin M. N., Karunanidhi A. (2013). Quantitative PCR analysis of genes expressed during biofilm development of methicillin resistant *Staphylococcus aureus* (MRSA). *Infection, Genetics and Evolution*.

[32] He Y. T., Du J. Y., Ma C. Y., Bai F. L. (2008). Analysis on antimicrobial activities of pollen polysaccharide in vitro. *Science and Technology of Food Industry*.

[33] Zhai Y. F., Zhang X. X., Xiang Q. S., Zhao L. X., He N. L., Shen R. L. (2019). Antimicrobial activity of pumpkin polysaccharide in vitro. *Food Research And Development*.

[34] Hu R. P., Ao C. J., Du L., Xue X., Deng F., Sun X. L. (2015). Study on the antibacterial activity of bletilla striata polysaccharide. *Anhui Agri Sci Bull*.

[35] Hu R. P., Ao C. J., Du L., Xue X., Deng F., Sun X. L. (2011). Research of bacteriostatic effect of allium mongolium regel poly saccharides in vitro. *Journal of Inner Mongolia University (Natural Science Edition)*.

[36] Sawangkan K., Sittikijyothin W., Satirapipathkul C. (2012). Natural polysaccharide-based films and their antibacterial activities. *Advanced Materials Research*.

[37] Zhao X. C. (2016). *Studies on the Mechanism of Anti-Glust Staphylococcus aureus Activity of Tea Tree Oil*.

[38] Pozo J. L. D. (2018). Biofilm-related disease. *Expert Review of Anti-Infective Therapy*.

[39] Veerachamy S., Yarlagadda T., Manivasagam G., Yarlagadda P. K. (2014). Bacterial adherence and biofilm formation on medical implants: a review. *Proceedings of the Institution of Mechanical Engineers, Part H: Journal of Engineering in Medicine*.

[40] Li X. F. (2012). *New Evaluation Methods for the Scavenging Effect of Plant Polyphenols on DPPH Free Radical and Their Application*.

[41] Reijer P. M. d., Haisma E. M., Toom N. A. L.-d. (2016). Detection of alpha-toxin and other virulence factors in biofilms of *Staphylococcus aureus* on polystyrene and a human epidermal model. *PLoS One*.

[42] Tang J., Nie J., Li D. (2014). Characterization and antioxidant activities of degraded polysaccharides from poria cocos sclerotium. *Carbohydrate Polymers*.

